# Supplementary figures and images for: The conserved protein adaptors CALM/AP180 and FCHo1/2 cooperatively recruit Eps15 to promote the initiation of clathrin-mediated endocytosis in yeast
Source: PLoS Biol. 2024 Sep 24;22(9):e3002833. doi: 10.1371/journal.pbio.3002833 (PMC11451990; doi:10.1371/journal.pbio.3002833)

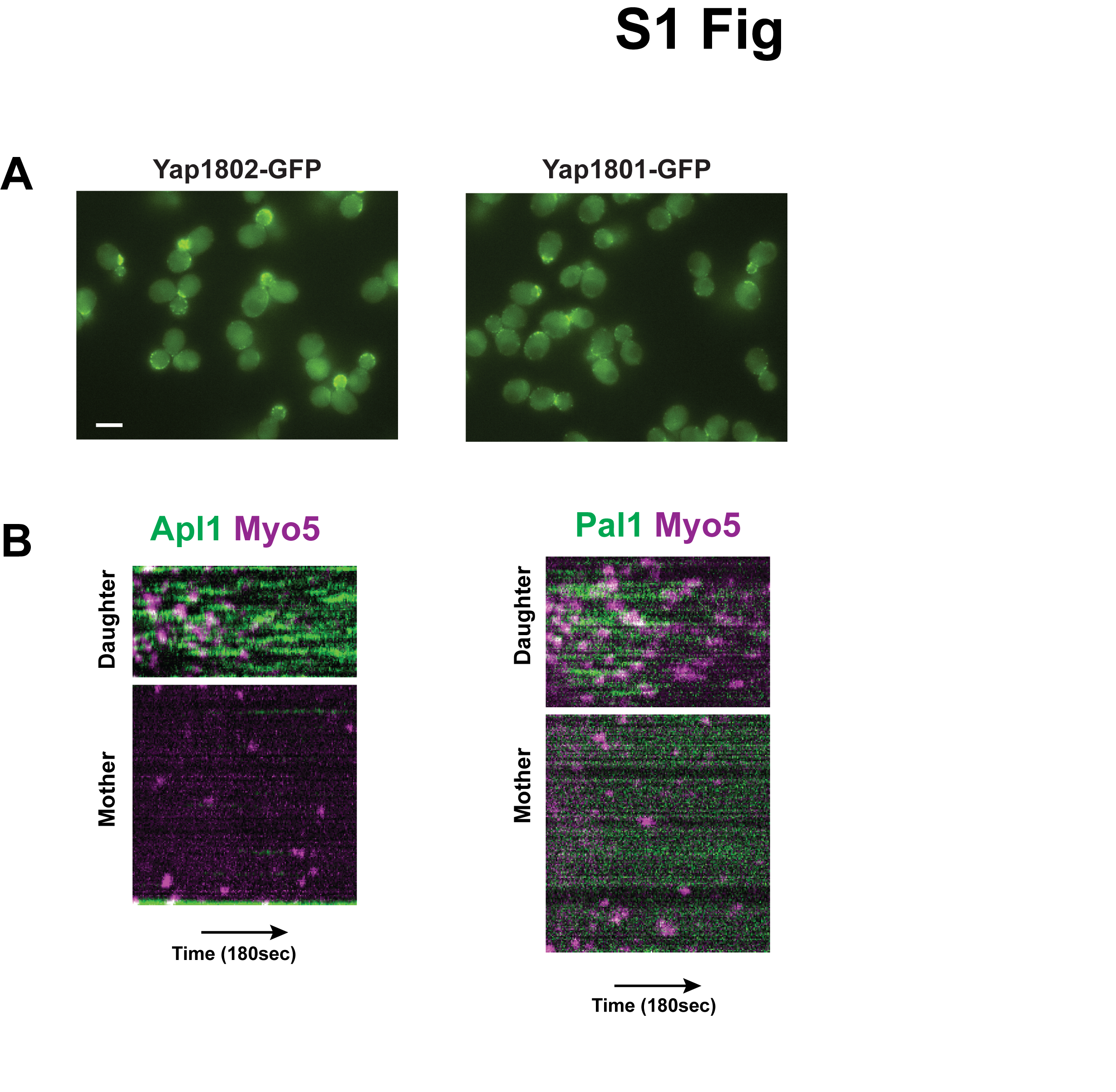

Supplement: S1 Fig — (A) Single images of cells endogenously expressing Yap1801-GFP or Yap1802-GFP. The 2 images were created using identical microscopy settings. The bar is 5 μm. (B) Two-color circumferential kymograph representations of mother and daughter cells endogenously expressing Apl1-GFP/Myo5-mScarlet-I or Pal1-GFP/Myo5-mScarlet-I. (TIF) [file pbio.3002833.s001.tif]

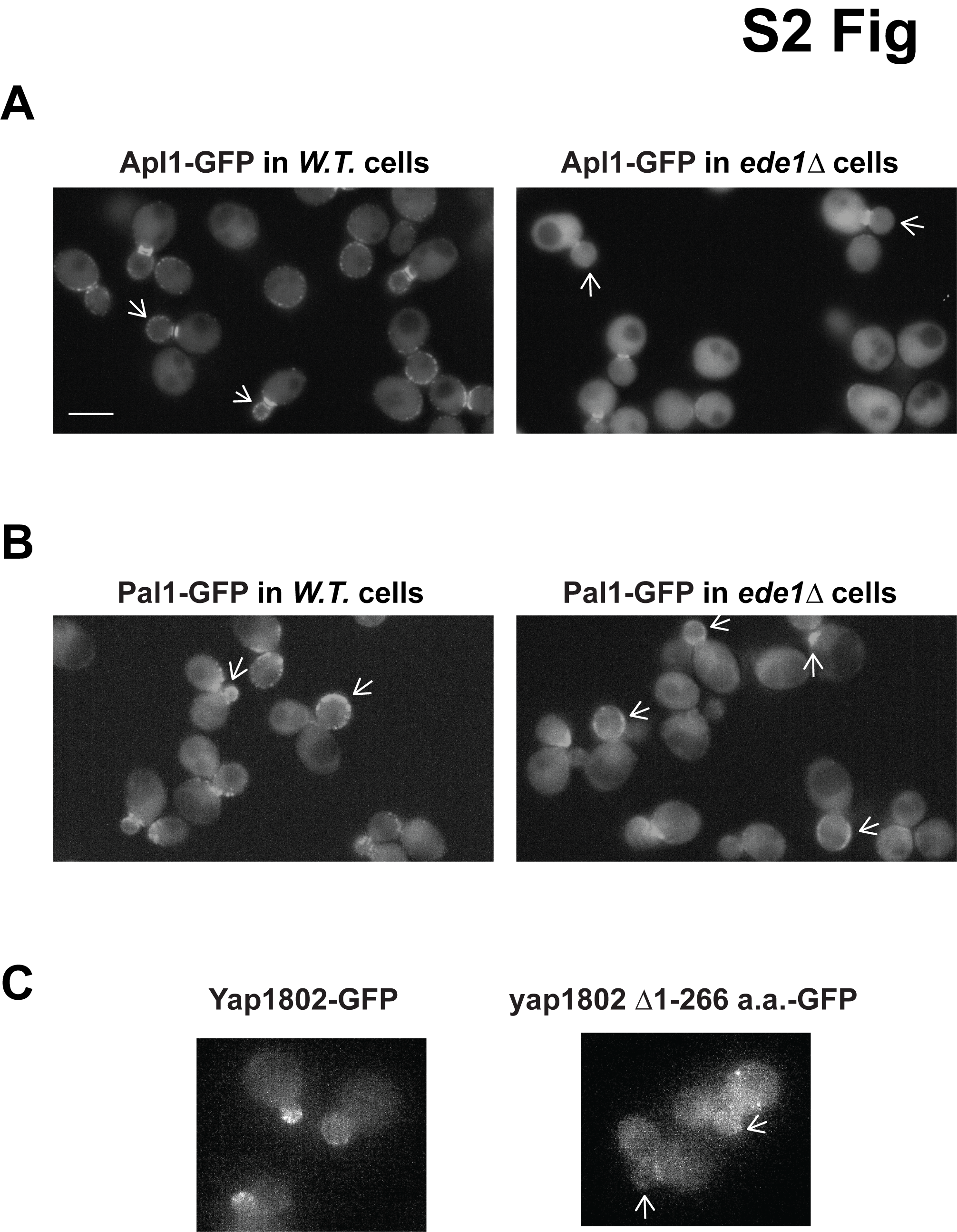

Supplement: S2 Fig — (A, B) Localization of Apl1 (A) or Pal1 (B) in wild-type or ede1Δ cells. Maximum intensity projections (MIP) of 3 min-movies were created for the indicated yeast strains. The arrows highlight the polarized localization of Apl1-GFP (A, left panel) and Pal1-GFP (B, left panel) in wild-type cells. The arrows indicate that polarized Apl1-GFP localization is lost (A, right panel) while the polarized Pal1-GFP localization (B, left panel) is retained in ede1Δ cells. (C). Localization of ANTH domain truncated-yap1802. The arrows indicate that yap1802 Δ 1–266 a.a.-GFP no longer localizes at the cortex of the daughter cell. The scale bar is 5 μm. (TIF) [file pbio.3002833.s002.tif]

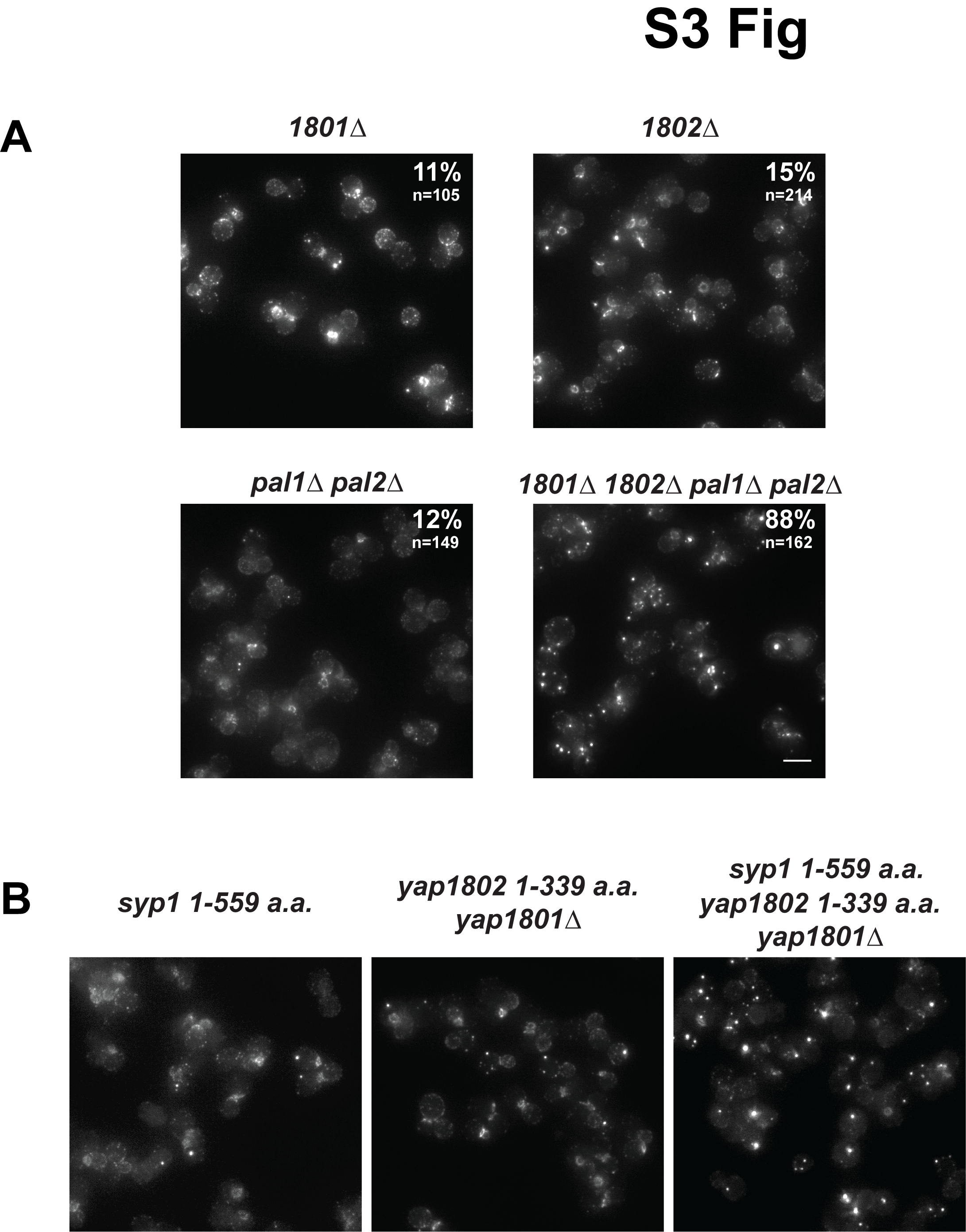

Supplement: S3 Fig — 3D Z-projection of Ede1-GFP (A) or Ede1-Scarlet-I (B) localization in the indicated mutants. The scale bar is 5 μm. (TIF) [file pbio.3002833.s003.tif]

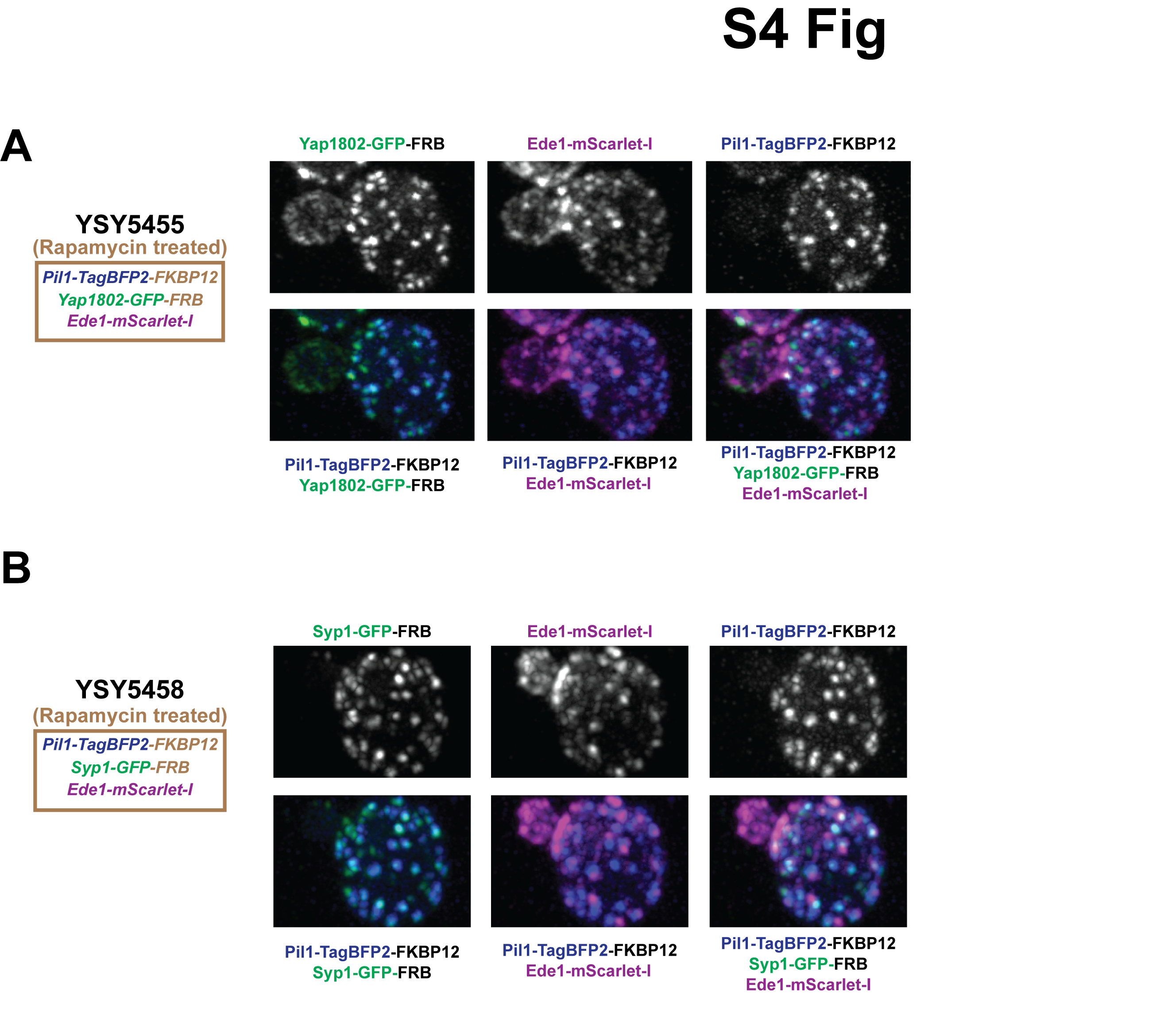

Supplement: S4 Fig — (A) or (B) represents detailed data for Fig 8C or 8D, respectively. (TIF) [file pbio.3002833.s004.tif]
