# Supplementary material for: The conserved protein adaptors CALM/AP180 and FCHo1/2 cooperatively recruit Eps15 to promote the initiation of clathrin-mediated endocytosis in yeast
Source: PLoS Biol. 2024 Sep 24;22(9):e3002833. doi: 10.1371/journal.pbio.3002833 (PMC11451990; doi:10.1371/journal.pbio.3002833)
Supplement: S1 Table — (PDF) [file pbio.3002833.s005.pdf]

Supplemental Table 1

**DDY904**	*MATα, ade2-1, his3Δ200, leu2-3,112, ura3-52, lys2-801*
**DDY1102**	*MATa/MATα, ade2-1/ADE2, his3Δ200/ his3Δ200, leu2-3,112/ leu2-3,112, ura3-52/ ura3-52, lys2-801/LYS2*
**YSY4456**	*mRuby2-TUB1::URA, SLA1-GFP::HIS*
**YSY4546**	*EDE1-GFP::LEU::HIS, MYO5-mScarlet-I::URA*
**YSY4593**	*SLA2-GFP::HIS EDE1-mScarlet-I::KAN*
**YSY4706**	*YAP1802-GFP::His, YAP 1801-GFP::KAN, MYO5-mScarlet-I::URA*
**YSY4609**	*PAN1-GFP::HIS SLA2-mScarlet-I::KAN*
**YSY4704**	*YAP 1802-GFP::HIS, YAP 1801-GFP::KAN, EDE1-mScarlet-I::URA*
**YSY4715**	*YAP 1802-GFP::HIS, MYO5-mScarlet-I::URA*
**YSY4974**	*SYP1-GFP::KAN EDE1-mScarlet-I::URA*
**YSY4968**	*syp1Δ::cgLEU EDE1-GFP::HIS*
**YSY4695**	*yap1801Δ::cgLEU, yap1802Δ::cgURA, EDE1-GFP::HIS*
**YSY4966**	*syp1Δ::cgLEU, apl1Δ::cgURA, EDE1-GFP::HIS*
**YSY4957**	*syp1Δ::cgLEU, yap1801Δ::NAT, yap1802Δ::URA, EDE1-GFP::HIS*
**YSY5462**	*yap1802Δ::HYG, yap1801Δ::NAT, syp1Δ::cgLEU, apl1Δ::cgURA, EDE1-GFP::HIS*
**YSY4765**	*apl1Δ::URA, yap1801Δ::NAT yap1802Δ::URA, EDE1-GFP::HIS*
**YSY4985**	*syp11-565A.A.-GFP:: HIS*
**YSY4998**	*yap1802 1-339A.A.-GFP:: HIS*
**YSY4846**	*MYO5-mScarlet-I::URA, SYP1-GFP::KAN*

YSY4847 *ede1Δ::cgLEU, MYO5-mScarlet::URA, SYP1-GFP::KAN*

YSY4848 *MYO5-mScarlet-I::URA, APL1-3GFP::HIS*

YSY4849 *ede1Δ::cgLEU, MYO5-mScarlet-I::URA, APL1-3GFP::HIS*

YSY4912 *ede1Δ::cgLEU MYO5-mScarlet-I::URA PAL1-GFP::HIS*

YSY4913 *MYO5-mScarlet-I::URA PAL1-GFP::HIS*

DCT128 *YAP1802-GFP::HIS*

YSY4573 *YAP1801-GFP::KAN*

YSY4848 *MYO5-Scarlet-I::URA, APL-3GFP::HIS*

YSY4913 *MYO5-mScarlet-I::URA PAL1-GFP::HIS*

YSY4628 *yap1801Δ::cgLEU EDE1-GFP::cdLEU::HIS PAN1-mScarlet-I::KAN*

YSY4629 *yap1802Δ::URA EDE1-GFP::LEU::HIS PAN1-mScarlet-I::KAN*

YSY4982 *yap1801Δ::NAT, yap1802Δ::URA, EDE1-GFP::HIS*

YSY5463 *pal1Δ::cgURA, pal2Δ::LEU, EDE1-GFP::HIS*

YSY5087 *pal1Δ::cgURA, pal2Δ::cgLEU yap1801Δ::NAT, yap1802Δ::URA, EDE1-GFP::HIS*

YSY5077 *yap1801Δ::cgLEU, yap1802 1-339 A.A.-GFP:: KAN, EDE1-mScarlet-I::URA*

YSY5464 *syp11-565A.A.-GFP:: HIS yap1802 1-339 A.A.-GFP:: KAN, yap1801Δ::NAT, EDE1-mScarlet-I::URA*

YSY2604 *ede1 EH*(W56A W176A W319A)-GFP ::LEU:: HIS*

YSY5041 *ede1 Syp1bindingΔ (1110AA-1247AAΔ)-GFP:: LEU:: HIS*

YSY5058 *ede1 EH*(W56A W176A W319A) Syp1bindingΔ (1110AA-1247AAΔ)-GFP:: LEU:: HIS*

**YSY5465**	*syp11-565A.A.-GFP:: HIS , yap1802 1-339 A.A.-GFP:: KAN, yap1801Δ::NAT, EDE1-mScarlet-I::URA,*
**YSY5467**	*ede1 Syp1bindingΔ (1110AA-1247AAΔ)-GFP:: LEU :: HIS, yap1802Δ::HYG, yap1801Δ::NAT*
**YSY4991**	*syp11-565A.A-GFP::HIS JJTY 1075 ede1 EH* mutant ::cgLEU EDE1-mcherry::KAN tagged*
**YSY5455**	*YAP1802-GFP-FRB-cgLEU2, Pil1-TagBFP2-FKBP12-HygCYC, EDE1-mScarlet-I::URA, tor1-1, fpr1Δ::NAT*
**YSY5458**	*SYP1-GFP-FRB-cgLEU2, Pil1-TagBFP2-FKBP12-HygCYC, EDE1-mScarlet-I::URA, tor1-1, fpr1Δ::NAT*
**YSY5502**	*Pil1-TagBFP2-FKBP12-HygCYC, YAP1802-GFP-FRB-cgLEU2, SYP1-FRB::KAN, EDE1-mScarlet-I::URA, tor1-1, fpr1Δ::NAT*
**YSY4688**	*YAP 180-2-GFP::HIS YAP 180-1-GFP::KAN*
**YSY4725**	*ede1Δ::cgLEU, YAP 180-2-GFP::HIS, YAP 180-1-GFP::KAN,*
**YSY4989**	*syp1 1-565A.A-GFP:: HIS EDE1-mcherry::KAN::LEU*

All the YSY strains are derived from DDY904 or DDY1102
