# Supplementary material for: The conserved protein adaptors CALM/AP180 and FCHo1/2 cooperatively recruit Eps15 to promote the initiation of clathrin-mediated endocytosis in yeast
Source: PLoS Biol. 2024 Sep 24;22(9):e3002833. doi: 10.1371/journal.pbio.3002833 (PMC11451990; doi:10.1371/journal.pbio.3002833)
Supplement: Attachment — Submitted filename: Response_to_reviewers_comments_08092024.docx [file pbio.3002833.s011.docx]

*Reviewers' comments

Rev. 1:
Sun and Drubin et al present a clever study in which they have studied the differences in endocytosis between mother and daughter budding yeast cells to gain insight into the impact of different imitator and adapter proteins on endocytosis. The author observe a marked difference in the number of successful endocytic events, with daughter cells have far more such events than mother cells. This differences is ascribed to the increase in negatively charged phospholipids and cargo proteins in developing daughter cells. Using this system, the authors present a quantitative analysis of various components of the core endocytic machinery including ede1, yap180, syp1, myosin, and components of the yeast homolog of AP2. Interestingly, the authors find that yap180 and syp1 work together to recruit ede1 to the membrane surface. Once this recruitment occurs, multivalent interactions between ede1 and other endocytic proteins drives endocytosis forward. The study is rigorously performed,
and the authors do a good job of putting their work in the context of open questions and past work. I think the paper is already of the quality level necessary for publication. I don't see the need for additional studies. I have only a few questions for the authors to consider as they prepare their manuscript for publication. These are more for my own curiosity rather than critical points.*

We very much appreciate the reviewer’s kind words. *1. When the authors mention that the daughter cell has more cargo proteins than the mother cell, why types of cargo molecules do they have in mind? How do these cargo molecules interact with the endocytic machinery? Are they likely to interact directly with yap180 vs. AP2, etc.? If so, how might the interactions impact interpretation of the results? I apologize if I have missed this in the discussion somewhere.*

We mention in the Discussion section that “Yap180s are adaptors for CME of the vesicle-associated SNARE protein Snc1 (*Burston* et al, 2008)” and that “Syp1 interacts with cargo including Snc1, Mid2, Ptr2 and Mep3 through DxY motifs (Apel et al, 2017)”. Currently, we are preparing a manuscript (Marchando and Sun et al, 2024), which provides evidence that v-SNAREs play an upstream role in CME initiation and, in concert with polarized anionic lipids, recruit AP180s to the daughter cell during cell growth. No changes were made to address this point.  *2. The authors finding that Syp1 is important for recruitment of ede1 to the plasma membrane is expected, based on recent and older literature. However, I was a bit surprised by the finding that yap180 recruits ede1. I had always thought of ap180/calm (homologs of yap1) as later arriving components. I believe this impression comes from the 2011 paper by Taylor and Merrifield (PLOS Biology), which puts Eps15/Fcho in the earliest group of proteins to arrive and CALM/Epsin/clathrin/ap2 in the second group, still arriving before dynamin. Are things different in yeast? Has it already been established in yeast that Yap180 arrives early? If so, perhaps this could be clarified in the manuscript (apologize if it's already there and I missed it.)*

In our earlier study we endogenously tagged CME-related proteins with fluorescent proteins and examined their dynamics, finding that Ede1, Syp1, AP-2, Yap180s, and clathrin arrive at the nascent endocytic site at approximately the same time (usually within 4 s) (Carroll et al, 2012). However, the fluorescence signal from these proteins is quite dim when they start to form into patches, precluding a definitive conclusion about whether there is a regular order of arrival. Our current study confirmed our previous results. While early-arriving endocytic proteins seem to appear at similar times, our live-cell imaging of various mutants strongly suggests that Syp1/FCHo (yeast/mammalian name) and Yap180/CALM (as well as the Pals) function upstream of Ede1/Eps15 in their cortical recruitment to establish endocytic sites. This type of analysis would be extremely hard to achieve in mammalian cells. Indeed, the previous studies in mammalian cells obtained inconsistent results about which proteins are critical for the initiation process. This is likely due to multiple technical and biological factors, including intrinsic flexibility and complexity in the initiation process itself, and difficulty in making comparisons between diverse cell types and physiological states. It is worth noting that most previous studies (including Taylor’s 2011 study) were done by analyzing fluorescent proteins expressed by transient transfection, while the endogenous proteins are still present. Our studies analyzed endogenously tagged proteins, expressed from their native genomic loci under control of their normal promoters. As the proteins and protein domains analyzed in our studies in yeast are conserved throughout eukaryotes, considerate might be worthwhile for others to perform similar analyses in mammalian cells in the absence of protein overexpression.

*3. Furthermore, if Yap180 indeed arrives early and is important for recruiting Eps15 (as the authors seem to show), then if may imply that snares are playing some role in initiation of endocytic sites? Specifically, as the authors mentioned, David Owen and others showed that AP180/calm are cargo adaptors for snares. What do the authors think about the implications of that in light of their own findings? Could it be that a requisite local concentration of snares is needed to recruit yap180 before eps15 can be recruited and endocytosis can go forward? Is it know whether there is a local increase in snare's in daughter cells? Could it be that this is one of the enriched cargos in the daughter cells? Would like to know your thoughts.*

The reviewer raises important points. As mentioned earlier in this response, we are, in fact, preparing a manuscript (Marchando and Sun et al, 2024), which provides evidence that v-SNAREs play an upstream role in CME initiation in yeast, and do so in concert with polarized anionic phospholipids, to recruit the AP180s to the daughter cell during cell growth. We demonstrate that endogenously GFP-tagged Snc1p (SNARE) accumulates at the plasma membrane in yeast daughter cells undergoing polarized growth, with some additional intracellular puncta, which may correspond to endosomal structures. Specifically, we show that Yap1802 mutants lacking Snc-binding or lipid-binding ability display a cortical recruitment defect. We hypothesize that the coincidence of cargo (SNAREs) and anionic phospholipids promotes yeast Eps15 recruitment by multivalent interactions through shared functional domains in yeast CALM/AP180 and FCHo to trigger endocytic site initiation. Also, we do cite work by David Owen and others. *All in all, this is a nice piece of work that uses a clever approach to get some greater clarity on how endocytosis is initiated in yeast, which raises interesting questions (as noted above) about how it might work in mammals. I recommend publishing the study without requiring revision.*
We very much appreciate the reviewer’s kind words about our study.

*Rev. 2:
In this study, the localization kinetics of several proteins involved in clathrin-mediated endocytosis (CME) was carefully examined in both mother and daughter compartments of early budded Saccharomyces cerevisiae cells. To quantitatively compare mother to daughter compartment protein localization intensities and lifetimes, a new image analysis pipeline was developed. This allowed the understanding of individual protein kinetics to be refined and subtle differences in kinetics and intensities revealed relative to previous descriptions. The analysis also validated that CME events are more abundant and regular in the growing and polarized buds and that not all sites of CME contain the full complement of CME proteins. Finally, the localization dependencies among early arriving proteins were examined leading to the conclusions that Syp1, Yap180s and Pal1 do not depend on Ede1 for polarized membrane localization and that Ede1 depends on multivalent interactions with the
aforementioned proteins for its cortical recruitment.*

*The descriptions of protein localization kinetics presented in this study are impressive and thorough, and the new image analysis pipeline may be useful to other groups studying cortical events and processes. The data are clearly presented, statistically sound, the experiments are logical, and the manuscript is also well-organized and written (with the exception noted below). It is a very solid contribution. However, the conceptual advance in understanding CME appears to be modest. Subtle differences with previous descriptions in localization kinetics of CME proteins have been identified. The work validates that CME is preferential to the growing bud and emphasizes the importance of Ede1 recruitment. Apparently, the details of Ede1 recruitment were not known previously but that Ede1 relies on multivalent interactions does not appear to be a major advance in understanding. I have only a few "major" comments for consideration and several minor.*
We are glad that Reviewer #2 thinks our study is a very solid contribution. We would like to take this opportunity to clarify/highlight the conceptual significance of our work. In Figures 1-4 of our study, we demonstrate that CME site formation in mother and daughter cells of budding yeast undergoing polarized growth exhibits several pronounced differences, which have not been described previously. These differences are (1) the frequency of site formation, (2) the dynamics of the full endocytic pathway, and (3) the protein composition of the CME sites. Ours is the first study to compare CME protein dynamics between mother and daughter cells thoroughly. Moreover, we combined quantitative, live-cell imaging with powerful yeast genetics to present novel evidence that Syp1/FCHo (yeast/mammalian name) and Yap180/CALM (as well as the Pals) function upstream of Ede1/Eps15 in their cortical recruitment to establish endocytic sites, while AP-2 and Ede1 exhibit interdependence in their cortical localization. These results can be explained by a simple model in which highly conserved Yap180/CALM and Syp1/FCHo are recruited to the cell cortex when they interact with anionic phospholipids and possibly cargos, and in which these proteins subsequently recruit Ede1/Eps15 to trigger endocytic site formation. It is worth noting that three groups recently proposed that Ede1 (Eps15 in mammalian cells) forms liquid-like protein condensates that catalyze CME site assembly in plants (Dragwidge et al, 2024), yeast (Kozak et al, 2022), and mammalian cells (Day et al, 2021). In this context, our results provide a mechanistic basis for understanding how Ede1/Eps15 condensates are restricted to the cell cortex even though this protein does not itself have a lipid-binding domain. Given that mammalian liquid-like Eps15 condensates have been implicated in providing an optimal catalytic platform for CME site formation, and that Ede1 is the Eps15 homologue in yeast (Lu et al, 2017), our proposed model is expected to be widely applicable.

*Major comments:

1. Instead of "yeast" or "budding yeast", please use Saccharomyces cerevisiae throughout the manuscript. For example, it is incorrect to state that Ede1 is the "yeast" name for Eps15. S. cerevisiae is not the only yeast used as a model organism for cell biology studies, not even the only budding yeast, and what is described here for S. cerevisiae (timing, protein concentration, etc. at CME sites) may not be generalizable to other yeast.*

We clarified that the budding yeast *Saccharomyces cerevisiae* is used for our studies in the Abstract, in the Introduction, and at the beginning of the Results section. We used budding yeast or yeast throughout the rest of the manuscript for simplicity.  *2. Portions of the discussion (for example, section beginning line 470) are repetitive with what is written in previous sections. Further attention to reducing repetitive elements and minimizing the re-stating of the results and introduction would improve it.*

We deleted the repetitive parts as suggested by the reviewer.  *3. This group has made numerous significant contributions to the understanding of CME. In a previous paper, Pedersen et al., 2020, it is stated "Thus, site maturation, rather than site initiation, accounts for the previously observed polarized distribution of actin patches in this organism." This statement seems to undercut the significance of studying CME initiation steps to explain differential CME in mother and bud compartments, particularly since the work presented here shows no difference in Myo5 or Sla1 kinetics. Thus, it would be helpful to the non-expert if the current work and model were discussed more fully in the context of previous work and models.*

The major goals and experimental approaches for this study and *Pedersen et al 2020* are different and the results are complementary. While *Pedersen et al 2020* compared CME site maturation at polarized mother versus depolarized mother cells, this current study compared CME site formation (including both initiation and maturation) between the daughters and mothers within budding yeast undergoing polarized growth. In part of our analysis, early and middle-arriving proteins that displayed longer and more variable lifetimes were observed in the mother cells for yeast undergoing polarized growth, consistent with previous observations (Pedersen et al 2020). However, more importantly, we now demonstrate for the first time that CME site formation exhibits pronounced differences between mother and daughter cells in yeast undergoing polarized growth while sharing the same pool of endocytic proteins. To the best of our knowledge, most of the endocytic proteins had never been analyzed in this manner. Our data demonstrated that productive CME events occur much more frequently and regularly in daughter cells compared to mother cells. Thus, with our new findings, we now support the perspective that polarized distribution of actin patches is at least partially due to the high CME site initiation rate in daughter cells. Following the reviewer’s suggestion, we discuss this point more thoroughly in the second paragraph of the first section of the Discussion.

*4. Syp1 is not included in the frequency or lifetime analyses presented in Figs 3 and 4B although it is included in the cartoons of 1A and 4A, and Figure 3H. It seems it is similar, if not identical to Ede1, and the data are apparently available so that information should be added to the text (frequency) and lifetime of 4B for completeness.*

We added this information in the text and Fig.4B as the reviewer suggested.
 *Figure comments:
1. Fig 1A. The paper includes work with Pal1/2 and therefore these should also be included in the timeline despite the fact that they are not apparently evolutionarily conserved.*

To keep the Fig 1A from being too busy, the recruitment timing of Pal1/2, AP-2, and clathrin are mentioned in the Figure 1 legend but not shown in the figure.

*2. Kymographs would benefit from a more detailed time axis - perhaps ticks at every 30 sec.*

We added a scale bar (30 sec) on the kymographs in Figure 2.

*3. Colorblind readers would appreciate a different dual color scheme, perhaps green/magenta rather than green/red.*

As the reviewer suggested, the color scheme is now changed from green/red to green/magenta in Fig. 3, Fig. 8, S1 Fig., and S4 Fig.

*Minor comments:
1. Line 177: duplicates information provided above for endogenous tagging of proteins.*

We have deleted the related sentence.

*2. Line 183: lifetimes*

Changed as suggested

*3. Line 197: Yap180s' GFP.*

Changed as suggested

*4. Verb tense is not consistent throughout the results section. Typically, results are presented in the past tense (as in line 145) rather than present tense (as in line 142). See again lines 158-161 for another example of switching verb tense.*

Corrected as suggested

*5. Line 212: mammalian*

Corrected as suggested

*6. Line 221: results do not conclude. This sentence needs to be re-worded.*

Changed to “these results indicate”

*7. Line 277: genotypes need to be italicized.*

Corrected as suggested

*8. Line 310: ditto to point 7.*

Corrected as suggested

*9. Line 424: this sentence needs to be re-worded.*

Re-worded for greater clarity

*10. Line 428: incomplete sentence.*

Changed.

*11. Line 242: mentions "over time". Would a single time point not show the same result? What is the advantage of including several time points - signal?*

The single time point did show a similar result. Yes, the advantage of presenting Maximum Intensity Projection (MIP) of a movie is to obtain a stronger signal.

*12. Line 262: please specify what previous results the current results are consistent with.*

Changed as the reviewer suggested.

*Rev. 3: Vytas Bankaitis – note that this reviewer has signed his review
This MS interrogates the mechanism by which the sites of CME initiation on the plasma membrane are determined. While this issue has been studied for decades, and there is general agreement in many areas, there remain questions/controversies regarding order of assembly, etc - particularly as these relate to the initiation phase of the process. The fact that a number of the proteins involved in the early stages of CME do not have obvious lipid-binding motifs contributes to the uncertainty.
Using the yeast system, the authors take advantage of the intrinsic asymmetry of acidic phospholipid and endocytic cargo molecule content (concentration) in newly formed daughter cells (relative to their mothers during polarized cell growth) to assess the relationship between cargo/anionic phospholipid densities, and the temporal recruitment of initiating proteins, as key properties of CME initiation sites. The authors deploy a powerful combination of quantitative imaging and genetic approaches to these ends. The 2D kymograph approach is novel and yields particularly important spatial and temporal information that is essential to the case being made. The conclusion is that CME initiation and maturation occurs at very different frequencies and rates in daughters vs mothers. The authors extend those conclusions to describe a pathway where Yap1801/1802 and Syp1 cooperate with cargo and lipids to launch yeast Eps15-driven CME assembly at those specific sites.

In summary, this is a clever and well-constructed MS that provides information that will advance our understanding of CME initiation and will do so significantly. The differences, and these are clear differences, in CME maturation and life time in daughter vs mother cells is quite extraordinary. There is little to quibble with regarding this well-presented and well-written story.*

Thanks very much for the kind words for our study.  *Minor suggestions for the authors and editor to consider (not to be taken as criticism) are as follows:
(i) The authors invoke cargo and anionic phospholipids in the intellectual construction they present. While this is certainly justified given what is known about CME, the MS would be strengthened by assessing the temporal recruitment of a cargo molecule. It would seem that the v-SNARES Snc1 or Snc2 would suit as their endocytosis would be required for support of the rapid plasma membrane growth in the newly born daughter. It seems to this reviewer that tracking such a reporter would also provide information as to 'productive' vs 'abortive' CME initiations.*

As an independent follow-up study, we have indeed investigated the dynamics and behaviors of the v-SNAREs Snc1 and Snc2 expressed at their endogenous loci. GFP-tagged Snc1p (SNARE) accumulates preferentially at the plasma membrane of yeast daughter cells undergoing polarized growth, with some intracellular puncta, which may correspond to an endosomal compartment. Currently, we are preparing a manuscript on these studies, which provides evidence that v-SNAREs play an upstream role in CME initiation, and, in concert with polarized anionic lipids, recruit the AP180s to the daughter cell during cell growth. Please also see our response to the 3rd point of Reviewer #1.

*(ii) The authors do not address the anionic phospholipid angle. The reviewer acknowledges that ts mutants in the inositol lipid kinases might be problematic. But, it would seem the FRB/FKBP system might be applied to wipe the PM of PtdIns(4,5)P2 or PtdIns4p. The 2D kymograph system would still permit conclusions even though the 'wipe' would likely be isotropic.*

We thank the reviewer for the suggestion of using the FRB/FKBP system to deplete PI(4,5)P2 or PI4P. Indeed, a similar experiment has been done to examine PI(4,5)P2 function in mammalian cells (Zoncu et al 2007). PI(4,5)P2 breakdown was achieved by the inducible recruitment of an inositol 5-phosphatase module to the plasma membrane through the rapamycin/FRB/FKBP system. PI(4,5)P2 depletion resulted in a dramatic loss of clathrin puncta, which correlated with a massive dissociation of endocytic adaptors from the plasma membrane in COS-7 cells. We suspect that similar results would be observed in yeast. We appreciate the suggestion and would like to perform this type of experiment in a future investigation.
